# Aberrant protein targeting activates quality control on the ribosome

**DOI:** 10.3389/fcell.2023.1198184

**Published:** 2023-06-06

**Authors:** Zemfira N. Karamysheva, Andrey L. Karamyshev

**Affiliations:** ^1^ Department of Biological Sciences, Texas Tech University, Lubbock, TX, United States; ^2^ Department of Cell Biology and Biochemistry, Texas Tech University Health Sciences Center, Lubbock, TX, United States

**Keywords:** signal peptide, signal recognition particle (SRP), ribosome, disease, protein targeting and transport, protein sorting, protein quality control, RNA degradation

## Introduction

Cells synthesize thousands of different proteins that should be delivered to different cellular compartments, integrated into membranes, or secreted outside of the cell to conduct their functions. Over 20 thousand genes are detected in a human genome including about 3,000 genes encoding secreted proteins and 5,500 genes encoding membrane proteins (Uhlen et al., 2015). Thus, about 40% of all proteins are transported through or integrated into cellular membranes. What happens to secretory/membrane proteins that are not able to be targeted to the endoplasmic reticulum (ER) because of the mutations in the signal peptides or defects in the protein transport machinery? These proteins are potentially harmful to cells if they are mislocalized. In this article we discuss secretory protein targeting, signal peptides interactions with transport machinery of the cells, defects in these processes, their possible implications in human diseases, and cellular mechanisms preventing synthesis of defective secretory proteins.

## Signal peptides and protein targeting to ER

**FIGURE 1:**
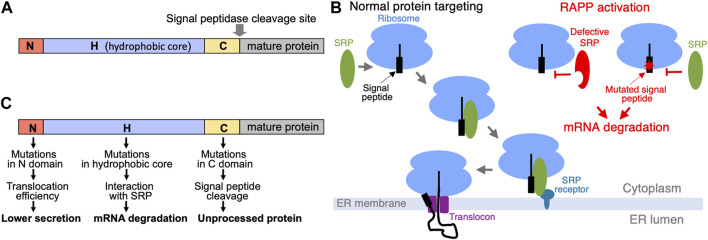
Signal peptides, protein targeting and its dysregulation. **(A)** Domain organization of signal peptides. **(B)** Scheme of SRP-dependent protein targeting in eukaryotes and Regulation of Aberrant Protein Production (RAPP) quality control on the ribosome. Under normal conditions SRP recognizes signal peptide and targets ribosomes to the SRP receptor in the ER membrane, and finally the secretory protein is translocated through the translocon to the ER lumen. However, when SRP is not able to recognize the signal peptide because of the mutations or because defects in the SRP itself, RAPP quality control is activated and the mRNAs are degraded. **(C)** Mutations in signal peptide domains affect different processes: mutations in the N domain affect efficiency of the protein translocation and secretion; mutations decreasing hydrophobicity of the H domain disrupt interaction with SRP, trigger RAPP protein quality control and lead to mRNA degradation of the mutated protein; mutations in the C domain may affect processing by a signal peptidase.

How cells distinguish secretory and membrane proteins from others to transport them? Many secreted proteins are synthesized with an extra N-terminal amino acid sequence called signal sequence or signal peptide that is removed (cleaved off) upon translocation of the proteins into ER lumen. These signal sequences are served as “tags” or “zip codes” to direct proteins to the ER for their further transport. Some membrane proteins also have cleavable signal peptides, while others use their first transmembrane spans for these purposes. Signal peptides from different secretory proteins do not have consensus of distinct amino acid sequences and they do not show significant amino acid homology. However, they have similar organization and three featured domains—N, H, and C (Figure 1A). The N is N-terminal portion of the signal peptides (1-5 amino acids), it often has one-two positively charged amino acids; H or hydrophobic core is presented by a region of 7-15 hydrophobic amino acids; and C region (3-7 amino acids) contains a site for signal peptidase (von Heijne, 1985; von Heijne, 1990). The signal peptide cleavage site is described by −3, −1 rule that provides restrictions of the amino acid residues in those positions of the signal peptide (von Heijne, 1983). Interestingly, signal peptides from different organisms (bacteria, yeast, mammals, etc.) have similar organization and features demonstrating universal concept for protein targeting signals.

Signal peptides are recognized by Signal Recognition Particle (SRP) (Hsieh and Shan, 2021; Kellogg et al., 2021). SRP dependent pathway is the major route for sorting of secreted and membrane proteins in mammalian cells. SRP is a complex of six proteins (SRP9, SRP14, SRP19, SRP54, SRP68, SRP72) and one noncoding RNA (7SL RNA) in mammals. SRP plays a key point in selecting proteins for their targeting to the ER membrane. SRP recognizes signal peptides of secretory protein precursors or membrane spans of membrane proteins immediately after their appearance from the ribosome during translation. SRP54 subunit directly binds signal peptides during translation. This interaction leads to the SRP molecular rearrangement, temporal elongation arrest, targeting complex to the SRP receptor in the ER membrane, transferring it to the translocon, then SRP leaves the complex, translation resumes, and the polypeptide nascent chain is co-translationally translocated into ER lumen through the translocon (Figure 1B). Proteins translocated to ER are folded with participation of the ER chaperones, subjected to co- and post-translational modifications and transported further.

## Aberrant signal peptides, defective SRPs, protein quality controls and human diseases

Mechanisms of protein targeting and transport are complex and, thus, many things can go wrong. It could be a problem with secretory protein itself because of genetic mutations or mistakes of transcription/translation, or issues with transport machinery because of defects in its components. Moreover, different stresses can affect proteins as well. These events can lead to protein mislocalization, misfolding and accumulation of insoluble protein aggregates that are potentially harmful. There are several cellular quality control mechanisms evolved to protect cells from these toxic species. They work at different levels and substrates, some of them are general and triggered by stress, others are specialized. Examples are responses to stress (unfolded protein response, UPR; ribosome-associated quality control, RQC, others), appearance of premature stop-codon mutations in mRNAs (nonsense mediated decay, NMD), mRNA truncations or ribosome stalling (no-go decay, NGD), or the absence of a natural stop codon (nonstop decay, NSD) (Brandman and Hegde, 2016; Shao and Hegde, 2016; Hetz and Papa, 2018; Joazeiro, 2019; Kurosaki et al., 2019; Sitron and Brandman, 2020; D'Orazio and Green, 2021). In addition, secretory and membrane proteins are controlled by a specific protein quality control on the ribosome termed Regulation of Aberrant Protein Production (RAPP) (Karamyshev et al., 2014; Karamyshev and Karamysheva, 2018). It senses signal peptide interactions with a targeting factor SRP and degrades the secretory protein mRNAs if these interactions are disrupted by mutations in a signal peptide or defects in SRP.

What mutations in signal sequences trigger RAPP? As mentioned above, the signal peptides have a domain organization. The domain structure of signal sequences reflects their functions. Thus, H region is crucial for binding with SRP54—deletions of hydrophobic amino acids from this area dramatically reduce interaction with SRP54, while alterations of charged amino acids in the N domain have only mild effect (Nilsson et al., 2015). Mutations in the signal peptide C region mostly affect processing (Karamyshev et al., 1998). However, impact of mutations in C region on SRP binding and protein transport have a minimal affect (Nilsson et al., 2015). Thus, hydrophobicity of the H domain is the major factor affecting SRP interaction with a signal peptide. Indeed, mutations decreasing hydrophobicity of the signal peptide in the preprolactin signal peptide H-region triggered degradation of the mutated protein mRNA (Karamyshev et al., 2014). Moreover, the effect of the mutations was graded—the mRNA degradation gradually increased with the severity of the mutations. Interestingly, depletion of the SRP54 subunit also triggers RAPP.

RAPP is a unique protein quality control—it co-translationally recognizes aberrant proteins at the ribosome and prevents their synthesis through specific mRNA degradation. It requires active translation. The RAPP response involves engagement of AGO2 protein, specific chaperone network (HSPA1A, DNAJB1, HSP90AA1, others), ribosome rearrangement through exchange of the ribosomal protein RPS27 and RPS27L, and ribosome-associated ubiquitination (Karamyshev et al., 2014; Tikhonova et al., 2022). It is proposed that ribosome heterogeneity and specialization may play an important role in many cellular processes (Genuth and Barna, 2018a; Genuth and Barna, 2018b; Miller et al., 2023). Thus, exchange of ribosomal proteins during RAPP activation may be important for its molecular mechanism. It is also established that RAPP is a general pathway controlling many secretory and membrane proteins (Tikhonova et al., 2022). However, details of the molecular mechanism of RAPP are not well understood.

There are many diseases associated with the defects in protein targeting to ER. Generally, they can be divided into two large categories—disorders associated with defects in a targeting factor SRP, and diseases associated with mutations in specific secretory proteins. The first category includes defects in SRP protein subunits and 7SL RNA leading to very diverse disorders including cancer, autoimmunity, hematological, neurological, neurodegenerative, infectious, and other diseases (Kellogg et al., 2022). The molecular mechanisms of these disorders are diverse and they depend on the affected subunit. The loss of SRP54 as a result of autoimmune response or some mutations in SRP54 may induce the RAPP quality control. Some mutations may interfere with the efficiency of SRP interaction with SRP receptor, or with SRP complex formation. Several studies demonstrated that mutations in the G-domain of SRP54 are associated with neutropenia and Shwachman-Diamond syndrome (Carapito et al., 2017; Bellanne-Chantelot et al., 2018; Juaire et al., 2021). The second category of diseases is associated with mutations in secretory proteins. It is connected with a loss of expression or a change of processing of a single secretory protein. Among them are frontotemporal lobar degeneration (FTLD) connected with granulin haploinsufficiency, aspartylglucosaminuria caused by defects in aspartylglucosaminidase, Wolman disease associated with mutations in lipase A, and many others (Jarjanazi et al., 2008; Karamyshev et al., 2020). Molecular mechanisms of these diseases are associated with location of mutations in secretory proteins and their severity. We proposed that mutations in the signal peptide H-domain decreasing hydrophobicity induce RAPP, while mutations located in the C-region do not activate RAPP, but instead may inhibit processing (Tikhonova et al., 2019) (Figure 1C). Mutations in the signal peptide N-domain are unlikely to induce RAPP because this area is not important for interaction with SRP, but it may be important for secretion efficiency. Recently we elucidated that a pathological RAPP activation and mRNA degradation of the granulins with W7R and A9D mutations is a molecular mechanism of FTLD pathology for the patients bearing these mutations (Pinarbasi et al., 2018; Karamysheva et al., 2019). The similar conclusions were made for many other secretory proteins with mutations that are associated with a number of human diseases (Tikhonova et al., 2019). Thus, pathological RAPP activation may play a significant role in many human diseases. Interestingly, the RAPP pathway is also involved in some cases of the Parkinson’s disease, however, the molecular basis of it is not clear yet (Hernandez et al., 2021).

## Conclusion

The protein transport is one of the most important cellular processes. About 40% of all proteins in a cell are secretory and membrane proteins. Mutations in the hydrophobic core of the signal peptide or depletion of SRP54 subunit of the SRP complex lead to the RAPP activation and elimination of the secretory protein mRNA template. Activation of the RAPP pathway can cause a number of human diseases; however, many details of the RAPP mechanism remain unclear such as what components of mRNA degradation machinery are involved in the degradation process, and how changes in ribosome composition/modification contribute to RAPP. Therefore, understanding of the fine details of the molecular mechanism of the RAPP pathway is crucial for the development of new strategies for treatment multiple human diseases.

## References

[B1] Bellanne-Chantelot C. Schmaltz-Panneau B. Marty C. Fenneteau O. Callebaut I. Clauin S. (2018). Mutations in the SRP54 gene cause severe congenital neutropenia as well as Shwachman-Diamond-like syndrome. Blood 132 (12), 1318–1331. 10.1182/blood-2017-12-820308 29914977 PMC6536700

[B2] Brandman O. Hegde R. S. (2016). Ribosome-associated protein quality control. Nat. Struct. Mol. Biol. 23 (1), 7–15. 10.1038/nsmb.3147 26733220 PMC4853245

[B3] Carapito R. Konantz M. Paillard C. Miao Z. Pichot A. Leduc M. S. (2017). Mutations in signal recognition particle SRP54 cause syndromic neutropenia with Shwachman-Diamond-like features. J. Clin. Invest. 127 (11), 4090–4103. 10.1172/JCI92876 28972538 PMC5663364

[B4] D'Orazio K. N. Green R. (2021). Ribosome states signal RNA quality control. Mol. Cell. 81 (7), 1372–1383. 10.1016/j.molcel.2021.02.022 33713598 PMC8041214

[B5] Genuth N. R. Barna M. (2018b). Heterogeneity and specialized functions of translation machinery: From genes to organisms. Nat. Rev. Genet. 19 (7), 431–452. 10.1038/s41576-018-0008-z 29725087 PMC6813789

[B6] Genuth N. R. Barna M. (2018a). The discovery of ribosome heterogeneity and its implications for gene regulation and organismal life. Mol. Cell. 71 (3), 364–374. 10.1016/j.molcel.2018.07.018 30075139 PMC6092941

[B7] Hernandez S. M. Tikhonova E. B. Baca K. R. Zhao F. Zhu X. Karamyshev A. L. (2021). Unexpected implication of SRP and AGO2 in Parkinson's disease: Involvement in alpha-synuclein biogenesis. Cells 10 (10), 2792. 10.3390/cells10102792 34685771 PMC8534902

[B8] Hetz C. Papa F. R. (2018). The unfolded protein response and cell fate control. Mol. Cell. 69 (2), 169–181. 10.1016/j.molcel.2017.06.017 29107536

[B9] Hsieh H. H. Shan S. O. (2021). Fidelity of cotranslational protein targeting to the endoplasmic reticulum. Int. J. Mol. Sci. 23 (1), 281. 10.3390/ijms23010281 35008707 PMC8745203

[B10] Jarjanazi H. Savas S. Pabalan N. Dennis J. W. Ozcelik H. (2008). Biological implications of SNPs in signal peptide domains of human proteins. Proteins 70 (2), 394–403. 10.1002/prot.21548 17680692

[B11] Joazeiro C. A. P. (2019). Mechanisms and functions of ribosome-associated protein quality control. Nat. Rev. Mol. Cell. Biol. 20 (6), 368–383. 10.1038/s41580-019-0118-2 30940912 PMC7138134

[B12] Juaire K. D. Lapouge K. Becker M. M. M. Kotova I. Michelhans M. Carapito R. (2021). Structural and functional impact of SRP54 mutations causing severe congenital neutropenia. Structure 29 (1), 15–28.e7. 10.1016/j.str.2020.09.008 33053321

[B13] Karamyshev A. L. Karamysheva Z. N. Kajava A. V. Ksenzenko V. N. Nesmeyanova M. A. (1998). Processing of *Escherichia coli* alkaline phosphatase: Role of the primary structure of the signal peptide cleavage region. J. Mol. Biol. 277 (4), 859–870. 10.1006/jmbi.1997.1617 9545377

[B14] Karamyshev A. L. Karamysheva Z. N. (2018). Lost in translation: Ribosome-associated mRNA and protein quality controls. Front. Genet. 9, 431. 10.3389/fgene.2018.00431 30337940 PMC6180196

[B15] Karamyshev A. L. Patrick A. E. Karamysheva Z. N. Griesemer D. S. Hudson H. Tjon-Kon-Sang S. (2014). Inefficient SRP interaction with a nascent chain triggers a mRNA quality control pathway. Cell. 156 (1-2), 146–157. 10.1016/j.cell.2013.12.017 24439374 PMC3931426

[B16] Karamyshev A. L. Tikhonova E. B. Karamysheva Z. N. (2020). Translational control of secretory proteins in Health and disease. Int. J. Mol. Sci. 21 (7), 2538. 10.3390/ijms21072538 32268488 PMC7177344

[B17] Karamysheva Z. N. Tikhonova E. B. Karamyshev A. L. (2019). Granulin in frontotemporal lobar degeneration: Molecular mechanisms of the disease. Front. Neurosci. 13, 395. 10.3389/fnins.2019.00395 31105517 PMC6494926

[B18] Kellogg M. K. Miller S. C. Tikhonova E. B. Karamyshev A. L. (2021). SRPassing Co-translational targeting: The role of the signal recognition particle in protein targeting and mRNA protection. Int. J. Mol. Sci. 22 (12), 6284. 10.3390/ijms22126284 34208095 PMC8230904

[B19] Kellogg M. K. Tikhonova E. B. Karamyshev A. L. (2022). Signal recognition particle in human diseases. Front. Genet. 13, 898083. 10.3389/fgene.2022.898083 35754847 PMC9214365

[B20] Kurosaki T. Popp M. W. Maquat L. E. (2019). Quality and quantity control of gene expression by nonsense-mediated mRNA decay. Nat. Rev. Mol. Cell. Biol. 20 (7), 406–420. 10.1038/s41580-019-0126-2 30992545 PMC6855384

[B21] Miller S. C. MacDonald C. C. Kellogg M. K. Karamysheva Z. N. Karamyshev A. L. (2023). Specialized ribosomes in Health and disease. Int. J. Mol. Sci. 24 (7), 6334. 10.3390/ijms24076334 37047306 PMC10093926

[B22] Nilsson I. Lara P. Hessa T. Johnson A. E. von Heijne G. Karamyshev A. L. (2015). The code for directing proteins for translocation across ER membrane: SRP cotranslationally recognizes specific features of a signal sequence. J. Mol. Biol. 427 (6), 1191–1201. 10.1016/j.jmb.2014.06.014 24979680 PMC4277940

[B23] Pinarbasi E. S. Karamyshev A. L. Tikhonova E. B. Wu I. H. Hudson H. Thomas P. J. (2018). Pathogenic signal sequence mutations in progranulin disrupt SRP interactions required for mRNA stability. Cell. Rep. 23 (10), 2844–2851. 10.1016/j.celrep.2018.05.003 29874572 PMC6097231

[B24] Shao S. Hegde R. S. (2016). Target selection during protein quality control. Trends Biochem. Sci. 41 (2), 124–137. 10.1016/j.tibs.2015.10.007 26628391

[B25] Sitron C. S. Brandman O. (2020). Detection and degradation of stalled nascent chains via ribosome-associated quality control. Annu. Rev. Biochem. 89, 417–442. 10.1146/annurev-biochem-013118-110729 32569528 PMC8258965

[B26] Tikhonova E. B. Gutierrez Guarnizo S. A. Kellogg M. K. Karamyshev A. Dozmorov I. M. Karamysheva Z. N. (2022). Defective human SRP induces protein quality control and triggers stress response. J. Mol. Biol. 434 (22), 167832. 10.1016/j.jmb.2022.167832 36210597 PMC10024925

[B27] Tikhonova E. B. Karamysheva Z. N. von Heijne G. Karamyshev A. L. (2019). Silencing of aberrant secretory protein expression by disease-associated mutations. J. Mol. Biol. 431 (14), 2567–2580. 10.1016/j.jmb.2019.05.011 31100385 PMC6684239

[B28] Uhlen M. Fagerberg L. Hallstrom B. M. Lindskog C. Oksvold P. Mardinoglu A. (2015). Proteomics. Tissue-based map of the human proteome. Science 347 (6220), 1260419. 10.1126/science.1260419 25613900

[B29] von Heijne G. (1983). Patterns of amino acids near signal-sequence cleavage sites. Eur. J. Biochem. 133 (1), 17–21. 10.1111/j.1432-1033.1983.tb07424.x 6852022

[B30] von Heijne G. (1985). Signal sequences, the limits of variation. J. Mol. Biol. 184 (1), 99–105. 10.1016/0022-2836(85)90046-4 4032478

[B31] von Heijne G. (1990). The signal peptide. J. Membr. Biol. 115 (3), 195–201. 10.1007/bf01868635 2197415

